# Relationship Between Bench Press and Iron Cross Maximal Isometric Contraction—How to Develop the Strength to Perform the Iron Cross on Rings

**DOI:** 10.1002/ejsc.70002

**Published:** 2025-07-10

**Authors:** Lecocq Tom, Gouelle Arnaud, Schärer Christoph, Mochizuki Luis, Tordi Nicolas

**Affiliations:** ^1^ Université de Reims Champagne‐Ardenne PSMS Reims France; ^2^ Fédération Française de Gymnastique Paris France; ^3^ Department of Elite Sport Swiss Federal Institute of Sport Magglingen (SFISM) Magglingen Switzerland; ^4^ School of Arts, Sciences and Humanities (EACH) University of São Paulo São Paulo Brazil

**Keywords:** assessment, injury & prevention, modeling, strength, training

## Abstract

The last code of point ruling international artistic gymnastic competitions took another step toward the necessity of strength difficulty on rings. The present study aims to analyze the relationship between bench press and iron cross maximal isometric contraction. 52 gymnasts (mean ± SD, 22.4 ± 5 years, 173 ± 5 cm, and 68.5 ± 6 kg) were randomly assigned to start by one of two exercises: (1) Maximal isometric contraction in the bench press position. The force applied by the athletes against an immobile bar was measured with handheld dynamometers. (2) Maximal contraction in the iron cross position. The participants were standing on force plates to measure their body weight at rest and during the maximal contraction. The force developed was computed by subtracting the remaining weight to the body weight. Both maximal forces were normalized by the body weight. Contrary to the expected relationship, bench press and iron cross are moderately correlated 0.41 (*p* = 0.003 and 95% IC [0.15; 0.61]). Rather than a linear trend, this study highlights a linear threshold between bench press and iron cross. Although some athletes present a high strength in bench press and a low strength in iron cross, it is noteworthy that no athletes present a low strength in bench press and a high strength in iron cross. This highlights that although bench press seems necessary, it is not sufficient for the iron cross. Based on the results, a model was developed to help coaches provide training recommendations established on the gymnast's current general and specific strength status.

## Summary

- Rather than a linear relationship between bench press and iron cross maximal isometric contractions, a linear threshold seems to determine the maximal theoretical force in iron cross depending on the force produced in bench press.
- A theoretical model helps coaches provide guidelines that depend on current general and specific strength status to improve the performance of the iron cross.
- No gymnasts presenting a low bench press, high iron cross relative strength highlights the necessities of the bench press for the iron cross, whereas the important number of gymnasts presenting high bench press, low iron cross relative strength emphasizes that although necessary, bench press is not sufficient for the iron cross.

## 1 Introduction

The last up‐to‐date code of points from the Fédération Internationale de Gymnastique took another step toward the ubiquitous necessity of strength elements for still rings. Indeed, requirements for both strength and hold element (Group II) and swing to strength hold elements (Group III) changed toward a larger reward for higher difficulty: “*Each Element Group requirement fulfilled by A, B, C, is awarded with 0.3 points by the D‐Jury*” while “*Each Element Group requirement fulfilled by D or higher element […] is awarded with 0.5 points by the D‐Jury.*” (Fédération Internationale de Gymnastique (FIG) 2025). The difference between an apparatus specialist and a gymnast performing A, B, or C element to validate the requirements is going to be larger than ever after these new Olympic cycle revisions. Strength and hold elements as well as swing to strength hold elements on still rings reward the athlete's ability to overcome the effect of gravity. The gymnast needs to generate a force at shoulder level equal to his body weight for hold elements (Fujihara 2023) whereas an even larger force is required during swing to hold elements (Merz et al. 2023). The biomechanical constraints on still rings are so critical that a form of selection is taking place. Apparatus specialists are consistently found among the smallest and the lightest gymnasts across multiple anthropometric studies (Atiković et al. 2017; Bagci 2023; Šibanc et al. 2017). Lighter and shorter gymnasts inherently present biomechanical advantages to be performant on rings.

According to the training pyramid described by Tudor Bompa, psychological, mental, tactical, and technical factors are built on the physical foundation (Bompa and Buzzichelli 2019) and the stronger the foundation, the greater the potential for developing the other attributes. In other words, the technical layer evaluated during competition will always be constrained by the physical foundation developed during training. Athletes need to be well physically conditioned to be able to perform the most rewarded elements according to the code of points. To achieve this aim, preconditioning exercises are fundamental in gymnastics. A large proportion of the still rings literature deal with the relation between physical preparation and still rings performance (Malíř et al. 2023). Hubner et al. analyzed the relation between preconditioning exercises and hold elements thanks to a special pulley system, where the body weight can be increased by additional weight or decreased by counterweight. Among the 8 gymnasts tested, almost 50% of the variability observed on the rings could be explained by the variability on the preconditioning exercises. They obtained a Spearman's correlation coefficient of 0.67 between 1RM bench press and the weight necessary to maintain an iron cross for 3 s (Hübner and Schärer 2015). Nevertheless, “*several studies aimed to strength and power development had already been published; however, it is not clear what preconditioning exercises and types of muscle contraction are preferred for selected strength elements and hold elements with maximal strength, relative strength or strength endurance.*” (Malíř et al. 2023). In addition to the paucity of information regarding the different conditioning exercise and the different muscle contraction type, Malir and colleagues also highlight the injury risk inherent to the still rings due to the unstable nature of the apparatus in conjunction with a large load at shoulder level (Brewin et al. 2000). Indeed, shoulders and hips are both ball and socket joints that allow an important range of motion. Contrary to the weight‐bearing purpose of the hip, the socket of the shoulder is smaller, allowing a greater range of motion for prehension purposes. However, the greater range of motion is obtained at the expense of a greater instability. In a 20‐year follow‐up at a national training center, not a single year has passed without at least one shoulder surgery. Out of the 30 shoulders oriented toward surgical treatment, 50% were injured on still rings (12 during traction in forced flexion‐rotation and 3 during isometric contraction against gravity). The remaining 50% are distributed between horizontal bar (9 shoulders) and parallel bars (6 shoulders). During the follow‐up, four gymnasts even underwent surgery on both shoulders (Gendre and Boileau 2021).

Maximal strength is paramount in sports and has been linked to performance. Despite the time‐consuming nature of testing, assessment and monitoring of the athletes' strength qualities is vital in the fast‐paced life of elite athletes. The use of isometric contraction instead of 1RM procedure is based on simplicity, time efficiency, minimal coordination, minimal injury risk, and reduced fatigue (Warneke et al. 2023). Despite acceptable coefficients of correlation between both test modalities, Warneke and colleagues express a warning regarding the fact that, for a large number of sports, isometric contraction does not constitute the main regimen, resulting in a lack of specificity of the testing protocol. However, hold elements on still rings are by definition quasi‐isometric contraction where both concentric and eccentric contraction occur to overcome gravity (Schärer et al. 2019, 2021). In addition to the abovementioned advantages about reduced time and injury risk, isometric testing also presents a higher ecological validity for male gymnasts evolving on rings compared to isotonic testing. A higher ecological validity could have a positive impact on the subjective face validity of the protocol, later increasing the interest and participation rate of gymnasts and coaches.

Despite the obvious discrepancies between bench press and iron cross movement in terms of body position, joint angles, and direction of the force, the correlation coefficient of 0.67 obtained by Hubner et al. nevertheless indicates an association between those two actions. As stated by Hübner and Schärer (2015), “*The strong correlations between the* Bench Press *exercise and all three ring strength elements (though only one was significant) were surprising, since* Bench Press *is rather unspecific at first glance […] the importance of the pectoral muscles for the holding elements on rings could help explain these relationships.*” The bench press exercise not only presents a good relationship with still rings performance but also an overall picture of the shoulder's status. A large number of muscles contribute to the different hold elements, both to perform and stabilize the position. On one hand, according to S. Bernasconi et al. (2004), the five muscles contributing mostly to the overall iron cross muscle activity are the biceps brachii (18.35% ± 2.63), the triceps brachii (17.86% ± 2.78), the teres major (15.76% ± 5.74), the pectoralis major (14.35% ± 2.22), and the latissimus dorsi (9.15% ± 2.44). On the other hand, for the bench press, according to the traditional muscle model, the primary movers are pectoralis major, triceps brachii, and deltoideus (anterior bundle). Some studies also suggest the latissimus dorsi as an additional mover (Stastny et al. 2017). Although correlation coefficient is not a cause–effect relationship, it might, however, suggest muscles contributing to both exercises.

Therefore, the objective of the present study is to analyze the relationship between bench press and iron cross maximal contraction in an isometric setup. Based on this relationship, a theoretical model will be provided to help coaches with individual and personalized training recommendations relying on the gymnast's current general and specific training status. The originality of the present work lies in the use of isometric contraction as opposed to isotonic contraction due to the specificity of ring's performance. Therefore, it is hypothesized that despite the aforementioned discrepancies between bench press and iron cross, the linear relationship between those two exercises could be stronger than what is currently available in scientific literature (i.e., Spearman's rho of 0.67 obtained by Hubner et al. in 2015).

## 2 Method

### 2.1 Participants

The protocol took place over 3 days during a national subelite competition. The all‐round champion obtained 77.400 points whereas the ring event champion obtained 13.300 points. Inclusion criteria were as follows: men, older than 18 years old, with a federal license and insurance, familiarized with both bench press and iron cross exercise, free of injuries, and with no upper‐limb injuries during the last 6 months. Out of the 55 participants who participated in the study, 52 were kept for further analysis (mean ± SD (standard deviation), age: 22.4 ± 5 years, height: 173 ± 5 cm, and weight: 68.5 ± 6 kg). One subject used his right to withdraw from participation at any time before the end of the protocol, and two participants' export files were damaged.

### 2.2 Procedures

**FIGURE 1:**
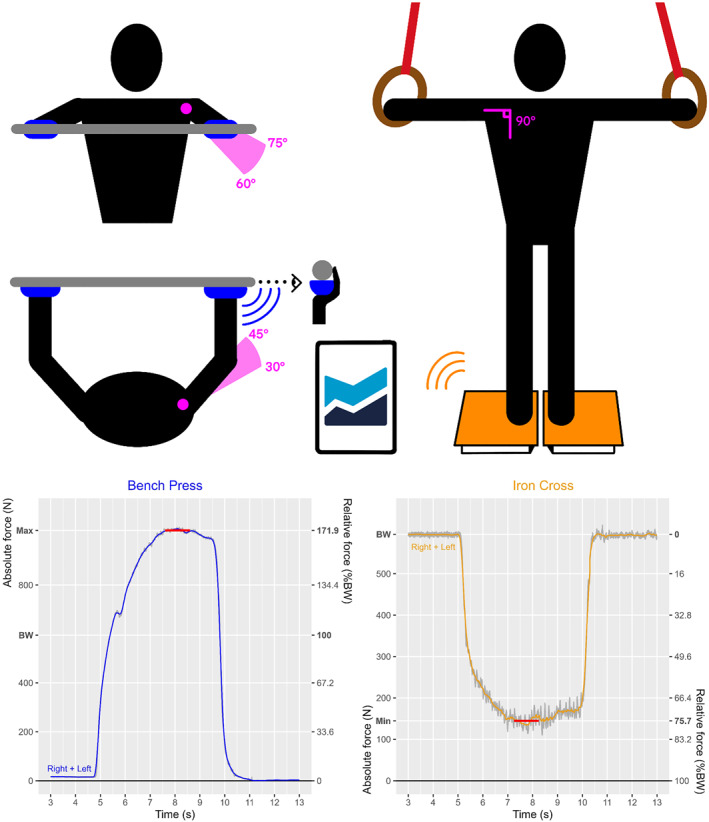
Protocol setup and data processing for bench press and iron cross exercises. The bench press signal (left) is equal to 0 at the beginning and at the end of the measurements since no force was applied (5 s of rest). During the five intermediate seconds, the force applied increase up to the gymnast's maximal force. The iron cross signal (right) is equal to the gymnast's body weight on both sides and decreases down in the middle to a minimal value obtained when the gymnast produced the maximal force downward.

Athletes received information about the protocol 1 month prior to the competition and were free to partake after signing an informed consent on the day of participation. The conformity of the protocol with the Declaration of Helsinki for research on human participants was validated by the national federation legal department. Warm‐up instructions and exercise description were reminded to each athlete before the protocol. After a 10 min warm‐up (i.e., planking, push‐ups, pull‐ups, and 30 kg bench press), participants were randomly assigned to start with one of the two exercises presented in Figure 1. One in every two participants started with the maximal isometric contraction in bench press position whereas the other started in iron cross position. For both exercises, two maximal isometric contractions were performed with standardized verbal encouragement. The sentence pronounced by the researcher was “Five, Four, Three, Two, One, GO, GO, GO, […], Stop”. Each recording was 15 s long with the contraction performed from the fifth to the tenth second (i.e., 5 s buffer at the beginning and at the end of the measurements). A second trial was attempted once the gymnasts felt adequately recovered (between 1 and 3 min of rest). Afterward, subjects had on average 5 min between the two different exercises corresponding to rest, change in exercise stations as well as instruction for the following task.

Exercise 1: Maximal isometric contraction in bench press position. Participants were lying on their backs with feet on the bench. Both hands of the gymnast were equipped with handheld dynamometers (K‐Push dynamometers, Kinvent Biomécanique SAS, Montpellier) to measure the force applied by the athletes against an immobile horizontal bar. The following instructions were given: “*Try to push the bar upward as hard as you can for 5 s*”. The contraction was performed with a very wide grip to decrease the elbow angle to a minimum and to target primarily the pectoralis major. The granularity (i.e., the size of the smallest changes) of the distance between the bench and the bar was 5 cm, resulting in small interindividual angle variation. Shoulder angles were visually inspected and varied between 60° and 75° in the frontal plane and between 30° and 45° in the transversal plane (Figure 1). Such angles in the transversal plane were chosen to prevent the pectoral from creating shear force on the glenohumeral joints (Ackland and Pandy 2009).

**FIGURE 2:**
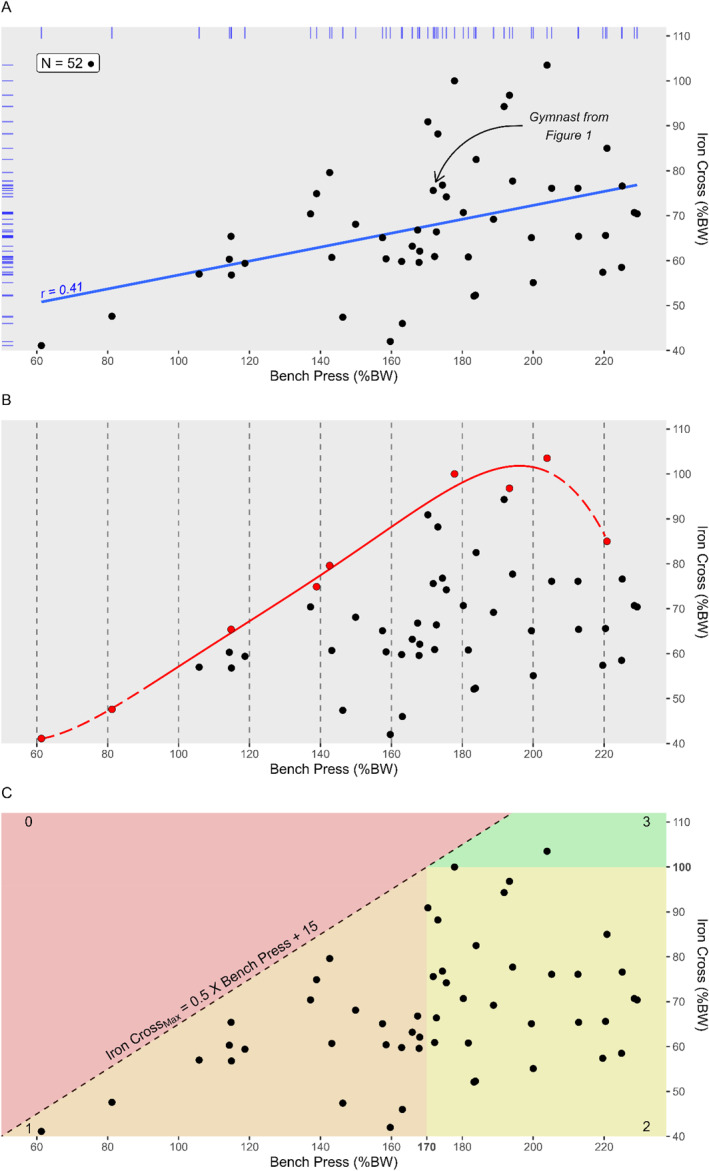
Relation between bench press and iron cross in terms of points (A), envelope (B), and zones (C). Dash dot plot A displays the best performance on each exercise for every individual. Plot B displays in red the best performers (i.e., highest iron cross force for every bench press 20% intervals) as well as the outside envelope of the population (i.e., red trend line). Plot C displays 4 different zones as well as a theoretical threshold computed from plots A and B. Zone 1 in orange presents the points below the threshold with a bench press relative force below 170%. Zone 2 in yellow presents the points with a bench press relative force higher than 170% but an iron cross relative force below 100%. Zone number 3 in green present the gymnasts with an iron cross relative force above 100%. No points are in zone 0 in red above the threshold.

Exercise 2: Maximal contraction in iron cross position. The participants were standing with each foot on a force plate (K‐Deltas, Kinvent Biomécanique SAS, Montpellier) that has been previously validated for static measurements (Mylonas et al. 2023). A pair of Gymnova rings with 3 m cables were hanging on each side of the gymnast at shoulder level (90° shoulder angle—horizontal arms). The following instruction were given: “*Try to lift your body for 5 s by pushing your hands downward as strong as you can while maintaining straight arms*”. Participants were allowed to touch the rings to be ready for the maximal contraction provided that they did not lift the rings (resulting in a larger weight) or let their arms rest in the rings (resulting in a lighter weight). The 5 s buffer at the beginning of the trial was used to measure the weight of the athletes (mean value between 0 and 3 s). In this setup, athletes able to perform the iron cross on official apparatus would take off from the force plates. In order to always keep the athletes in contact with the force plates, even though they are able to generate a force larger than their own body weight, an additional weight of 10 kg was placed in a backpack on their shoulder. The weight and the backpack were weighed before the protocol on the force plates and later subtracted to the force signal. Due to random sampling, among all the voluntary participants only one athlete was able to perform the iron cross on official apparatus, resulting in a single point above 100% BW relative strength on Figure 2. The choice was made to include him in the study despite the difference with the rest of the population. Although the bench press relative strength of athletes able to perform the iron cross is interesting, it is the analysis of the bench press relative strength of numerous gymnasts unable yet to perform the iron cross that allows drawing a path to successfully perform the iron cross.

Official still rings properties and dimensions are properly described: the distance from the point of attachment to the lower inner side of the rings should be 300 cm ± 1 cm long (FIG Apparatus Norms 2023; FIG Apparatuses Testing Procedures 2023). In this protocol, the cable length could vary in an interval of ± 5 cm compared to the official dimension so that all gymnasts had horizontal arms for the maximal contraction. However, for a gymnast with an arm span of 170 cm (Lehmann et al. 2021), the angle between the vertical and the cable calculated using Equation (1):

(1) $${\text{Angle}=\sin}^{-1}\left(\frac{\frac{\left(\text{Arm}\;\text{span}-50\right)}{2}}{\text{Cable}\;\text{length}}\right)$$

This angle was, respectively, 11.74° for 295 cm and 11.35° for 305 cm. The cables were long enough that a 10 cm length difference would result in a negligible angle variation (i.e., less than a degree).

### 2.3 Data Processing

Data processing and data visualization were performed on R (version 4.4.0/2024‐04‐24) with RStudio (IDE version 2024.04.2) thanks to the tidyverse library (Wickham et al. 2019). Right and left sensors signals were captured synchronously on a tablet (iPad Air (5th generation), Apple, Cupertino, California, USA) with the manufacturer software (Kinvent Physio, Kinvent Biomécanique SAS, Montpellier) at 1 kHz sampling frequency. Right and left sensors' signal were smoothed with a moving average (100 ms window) and summed together (Right + Left). The mean maximal force during bench press (Figure 1—Left) was computed as the mean value from 0.5 s before to 0.5 s after the maximal force (1 s window—Mean of 1000 points). The left axis represents the absolute force in Newton whereas the right axis represents the relative force normalized by the body weight in percentages. The mean maximal force during iron cross (Figure 1—Right) was computed as the mean value from 0.5 s before to 0.5 s after the center of the contraction (1 s window—Mean of 1000 points). The relative force on the right axis is in reverse order since when the force plate records the body weight, no force is produced, and when the force plate records zero, the gymnast has lifted his body and generated 100% relative force.

The maximal force was normalized by the athlete's body weight (i.e., measured on the force plates during the 5 s of rest at the beginning of the iron cross exercise) thanks to the following equations:

(2) $$\text{Bench}\;\text{Press}\;\left(\%\text{BW}\right)=\frac{\mathrm{Max}}{\text{BW}}\times 100$$

(3) $$\text{Iron}\;\text{Cross}\;\left(\%\text{BW}\right)=\frac{\text{BW}-\mathrm{Min}}{\text{BW}}\times 100$$

Out of the entire sample, the focus was placed on the best performers to better understand the relationship between bench press and iron cross relative strength. For every fixed interval on the bench press relative strength, solely the gymnast with the highest iron cross relative strength was used to unfold the outside envelope of the population. After multiple trials and errors, a 20% interval (i.e., [60%; 80%], [80%; 100%], [100%; 120%], etc.) presents the best empirical compromise between the number of intervals and the number of gymnasts present within the different intervals. Additionally, absolute individual variation between first and second trials on the bench press ranged between 0.1% and 9.7% (mean ± SD, 3.0 ± 2.6). The 20% interval therefore properly draws the outside envelope of the population while ensuring that gymnast could stay in the same interval despite biological variability.

### 2.4 Statistical Analysis

Intertrial reliability was assessed through intraclass correlation coefficient (ICC2,1—Two‐way random effects, absolute agreement, and single rater/measurement). According to recommendation, the ICC 95% confidence interval (CI) was compared to the following guideline: 0.00 ≤ ICC < 0.50 indicates poor reliability, 0.50 ≤ ICC < 0.75 indicates moderate reliability, 0.75 ≤ ICC < 0.90 indicates good reliability, and ICC ≥ 0.90 indicates excellent reliability (Koo and Li 2016). The normality of the distribution was verified with the Kolmogorov–Smirnov test (Gupta et al. 2019), and Pearson's correlation coefficient (r) was calculated with corresponding 95% CI to quantify the strength of the relationship between the two relative strength. Mean, SD, median and interquartile range (IQR) were computed to better describe the distribution. For all statistical analysis, the alpha level was set at 0.05.

## 3 Results

Bench press ICC is equal to 0.84 (*p* = 8.54e‐17 and 95%IC = [0.69; 0.92]); according to the guideline for interpreting ICC, the level of reliability can be regarded as “moderate” to “excellent”. The iron cross ICC is equal to 0.91 (*p* = 6.40e‐23 and 95%IC = [0.84; 0.95]), and the level of reliability can be regarded as “good” to “excellent”. For both exercises, test–retest reliability highlights the unlikely involvement of either a learning or a fatiguing effect between trials. Based on those results, the best of the two contractions was saved for further analysis (i.e., the highest force produced). Figure 2 presents the relationship obtained between relative strength in bench press and iron cross position. The suspicion of normal distribution that can be perceived from the density on the dash dot plot is confirmed by parametric testing for both variables (i.e., *p* = 0.75 and *p* = 0.57, respectively, for iron cross and bench press distribution). The maximal relative force in bench press ranged between 61.3% and 229.3% (Mean ± SD: 171.4% ± 37.5%). The median was 172.9% with half of the population between 155.6% and 195.5% (IQR). In the iron cross position, the relative force varied between 41.1% and 103.5% (Mean ± SD: 67.9% ± 14.2%) with a median equal to 65.5% and half of the sample between 59.6% and 76.1% relative force. Only one gymnast applied more than 100% BW in the iron cross position. Pearson's correlation coefficient between the bench press and the iron cross was 0.41 (*p* = 0.003 and 95%IC [0.15; 0.61]). Additionally, by focusing on the best performers (i.e., individuals in red for each column), the relationship between bench press and iron cross is fairly linear between 60% and 210% bench press relative strength. The center part of the outside envelope of the population (i.e., aligned with the belly of the normal distribution), therefore presents more certainty due to the larger number of gymnasts. Consequently, both right and left extremities of the envelope are traced with dashed line to symbolize said uncertainties.

## 4 Discussion

### 4.1 Relationship Between Bench Press and Iron Cross

This study examined the relationship between maximal isometric relative strength in the bench press position and in the iron cross position among subelite athletes. Contrary to the initial hypothesis, the linear relationship between the two relative strength is only moderated (*r* = 0.41, *p* = 0.003, and 95%IC [0.15; 0.61]). As it can be seen in Figure 2B, there exists a wide range of relative forces in the iron cross position for gymnasts with approximately the same relative strength on the bench press. If the relationship between bench press and iron cross was linear, all the gymnasts would be distributed between the lower left quadrant (Low–Low) and the upper right quadrant (High–High). Therefore, the bench press alone cannot be seen as a strong explanatory or predictor variable of the iron cross performance on the rings. Although some athletes are in the lower right quadrant (high in bench press but low in iron cross), it is noteworthy that no athletes are present in the upper left quadrant (low in bench press but high in iron cross). This highlights that although bench press seems necessary for the iron cross, it is not sufficient and that it is not possible to be strong in iron cross and weak in bench press. Instead of a linear relationship, there rather seems to be a linear threshold, highlighted in Figure 2C. The different zones around the threshold allow a new understanding of the interaction between bench press and iron cross.

The results of the present study, namely, the presence of a linear threshold instead of a linear trend between bench press and iron cross seems to be, at first glance, in contradiction with the results obtained by Hübner and Schärer (2015), where a Spearman's correlation coefficient of 0.67 was found between 1RM bench press and the weight (counterweight or additional weight) necessary to hold the position 3 s. The moderate correlation between the two relative strengths might be explained by the discrepancies between the two exercises evoked in the introduction (i.e., body position, joint angles, and force direction). Indeed, the bench press evaluates predominantly the strength of the pectoralis major and the triceps brachii. Although being activated by both movements, those two muscles are part of a larger number of muscles involved in a complex intermuscular coordination during the iron cross. In addition, the divergences with the results obtained by Hubner et al. in 2015 could also be explained by the characteristics of the population in terms of sample size, training content, and level of expertise. The current sample is composed of 52 subelite athletes, with only one athlete able to perform the iron cross, while the sample of Hubner et al. was formed by 8 top‐level male athletes from the Swiss national team, all able to hold the iron cross for 3 s, several with additional weight (mean additional weight: 3.3 ± 3.1 kg). The characteristics of the participants in age, height, and weight can be seen as a testimony of the differences between the two populations (i.e., 22.4 ± 5 years, 173 ± 5 cm, and 68.5 ± 6 kg for the present study v.s., 21.5 ± 2.5 years, 168.6 ± 4.5 cm, and 65.0 ± 5.0 kg for Hubner et al.). National teams are composed of the best performers nationwide; therefore, instead of a linear relationship, it is likely that Hubner et al. observed 8 athletes all pressing up against their individual theoretical maximal value. Iron cross relative strength can seemingly take any value between zero and the maximal theoretical value calculated thanks to Equation 4. The linear relationship obtained by Hübner et al. might instead represent each individual relative strength in the iron cross position maximized depending on the relative strength in the bench press position. It is possible to recalculate the individual relative strength on bench press of their sample. Percentages ranged between 145% body weight for athlete n°6 (body mass = 65.1/bench press = 95 kg) and 205% body weight for athlete n°8 (body mass = 55.9 kg/bench press = 115 kg). Despite the differences between dynamic and isometric contraction (Warneke et al. 2023), it can be seen in Figure 2B, that the relationship is also fairly linear within that range for the best performers nationwide present during this particular competition (i.e., individuals in red).

(4) $$\mathrm{Iron}\;\mathrm{Cros}s_{\max}=0.5\;\times \;\mathrm{Bench}\;\mathrm{Press}+15$$

### 4.2 Theoretical Model to Improve the Iron Cross

**FIGURE 3:**
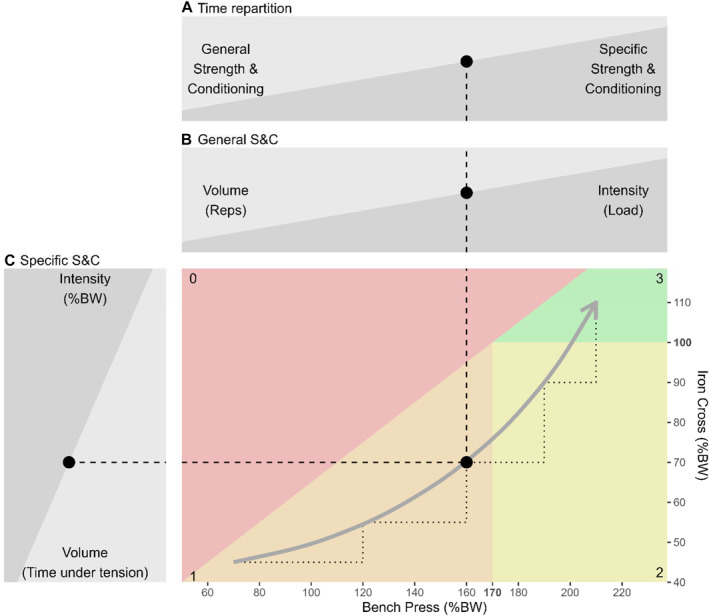
Theoretical model to improve iron cross.

Figure 3 presents a theoretical model to develop the strength required to perform the iron cross. The model is based on the premise that the current athlete's status regarding bench press and iron cross relative force is used as an input variable for the planification of the upcoming strength and conditioning cycle. Since the performance in the iron cross position seems to be conditioned by the performance in the bench press position (see threshold on Figure 2C), it is the bench press relative strength that is going to be the variable responsible for time distribution (Figure 3A) between general strength and conditioning (Figure 3B, to shift the gymnast rightward) and specific strength and conditioning (Figure 3C, to shift the gymnast upward). Once the time has been distributed, the volume and intensity are once again based on the gymnast's current performance. The model presented here is established on a “classical” or “linear” periodization for both general and specific strength and conditioning. At the beginning, the volume is important and the intensity is low to prepare muscles and tendons. Later, according to the volume/intensity trade‐off, the volume gradually decreases in favor of the intensity to develop maximal strength (Bompa and Buzzichelli 2019).

As explained by Schärer et al. (2021): “*combining conditioning strength tests with ring specific strength tests could provide important information about how much conditioning strength an athlete gained and how an athlete is able to apply this strength when performing strength elements on rings. This could help direct training on either developing more conditioning strength or on learning how to apply the force during hold elements (*“*technical*” *strength training of the static positions on rings).*” This notion of alternation between general and specific strength and conditioning is displayed as dotted stairs on Figure 3. The aim of the general strength and conditioning part is to develop the muscles and to improve the strength of the athletes (i.e., shifting rightward). The volume corresponds to the number of repetitions and sets whereas the intensity corresponds to the load. Those two variables are already easily manipulated by coaches during training. The aim of the specific strength and conditioning part is to apply more efficiently the strength previously gained by increasing the time spent on the rings (i.e., shifting upward). The volume is now “time under tension in isometric contraction” in seconds and the intensity is body weight percentage (Figure 3).

Various methods are already present in the literature and well‐known by coaches to manipulate those two variables:

The use of Herdos is a common method to decrease the mechanical stress on the shoulder by reducing the lever arm and therefore the torque at shoulder level (S. Bernasconi et al. 2004). Indeed, according to the same study, its usage yields a reduction in the normalized EMG signal for all 9 following muscles: pectoralis major, latissimus dorsi, teres major, infraspinatus, rhomboideus, trapezius, serratus anterior, biceps, and triceps brachii. However, the decrease is not proportional for all muscles. Specifically, the teres major muscle is the only one with a decrease that is not significant, resulting in a larger relative participation in the overall muscle's contribution with the Herdos than without. Herdos constitute a valid method to decrease shoulder torque and provide a smooth evolution thanks to various length settings; however, coaches need to be careful since its usage seems to change the shoulder's muscle synergy during the iron cross.

Furthermore, the use of Herdos with additional weight to match the torque generated on official apparatus could be an interesting intermediate step to protect the shoulders and the elbows (Marina et al. 2023). For all seven investigated muscles (pectoralis major, latissimus dorsi, teres major, trapezius, serratus anterior, biceps, and triceps brachii), the normalized EMG signal obtained with the additional weight was systematically between the official apparatus and the Herdos condition.

The belt (dream machine) is a different method that also decreases the mechanical stress on the shoulder but through the discharge of the load and not the decrease of the lever arm. Bernasconi et al. compared the muscle activity of 11 muscles (latissimus dorsi, serratus anterior, rhomboid, pectoralis major, infraspinatus, supraspinatus, seltoid (3 parts), biceps, and triceps brachii) during the Azarian (i.e., a slow roll backwards with straight body to iron cross) (S. M. Bernasconi et al. 2006). Regarding the last part of the movement, that is, the iron cross, larger normalized EMG signals were observed at the biceps brachii and at the middle bundle of deltoideus to stabilize the glenohumeral joint and at the triceps brachii to prevent upward dislocation of the humeral head and to keep the elbow joint into extension. The authors conclude that the belt could be used after the initial training with Herdos since it might prepare the shoulder joint more specifically and possibly induce closer muscles synergism. Moreover, although the belt corresponds to a cross performed with 50% of the body weight since ring cables are attached directly to the waist of the gymnast through pulleys, it is also possible to use a counterweight to modulate the percentage of body weight lifted by the gymnast and therefore vary the time under tension (e.g., 3 s, 5 s, and 7 s prediction) (Schärer and Hübner 2016).

Coach manipulation is another method that is used to decrease the force generated by the gymnast. However, engineering work will be required to “*objectively quantify the amount of spotting that a gymnast received as external physical assistance*” (Fujihara 2023).

Finally, other methods exist, such as the use of elastics, placed either between the rings or under the gymnast or training on a trolley/cart machine, which allows training all intermediate values between 0%BW and 100%BW by changing the angle of the frame from horizontality to verticality. If the friction of the trolley/cart is negligible, the load as a body weight percentage can be calculated from the sinus of the angle:

(5) $$\text{Load}\;\left(\%\text{BW}\right)=\frac{\text{Body}\;\text{Weight}\times \sin\left(\text{Angle}\right)}{\text{Body}\;\text{Weight}}\times 100$$

Despite the lack of literature concerning those last two methods, both were reportedly used in the preparation of Brazilian gymnast Arthur Zanetti before winning the still rings specialist Olympic Gold Medals in London 2012 (Goto et al. 2022)

## 5 Practical Applications

All the methods presented above consist in modifying the actual performance as little as possible in order to allow a large number of repetitions and a longer time under tension in the exact same position (i.e., shoulder adduction with straight arms). However, acknowledging the anatomical instability of the shoulder, the unstable nature of the apparatus as well as the magnitude of the stress applied on the rings, coaches might therefore be interested in training strategies that allow the strengthening of the muscles involved in the performance while also protecting the joints involved. It is likely that training on the rings will improve the strength of the muscle involved in the iron cross and the performance on the bench press; however, it implies a higher risk for the shoulders due to an important number of repetitions with straight arms and significant torque (Gendre and Boileau 2021).

The theoretical model is of great practical application since it allows coaches to pinpoint all gymnasts no matter what their initial level in terms of general and specific conditioning. It also allows to reveal individual strengths and weaknesses and to provide personalized orientations for improvements. Figure 3 displays in red the area above the threshold, which highlights that athletes first need to increase their bench press performance (i.e., shift rightward) to later be able to improve the iron cross (i.e., shift upward). According to Figure 3, it is only once gymnasts can produce 170% relative strength in bench press (0.5×170+15=100) that they might be able to perform the iron cross (green area under the threshold but above 100% relative force in iron cross). The fictional gymnast presented in Figure 3 is currently in zone 1 (160%; 70%). In order to perform the iron cross (e.g., gray arrow leading to zone 3), the gymnast will need to improve both his bench press and iron cross relative strength. The gymnast's current maximal theoretical relative strength in iron cross is 95% (i.e., 0.5×160+15), preventing a successful performance of the difficulty. The bench press relative strength allows distributing the time of training between general and specific strength and conditioning. The gymnast first needs to transition from zone 1 to zone 2 by improving the bench press relative strength. Secondly, transition from zone 2 to zone 3. It is only once the bench press relative strength is above 170% that a gymnast could produce an iron cross relative strength above 100% and successfully perform the element. If the gymnast is not able to transition from zone 2 to zone 3 (i.e., shift upward), it might highlight a lack of specific strength and conditioning in order to convert strength into technical strength applicable on the rings or a disharmonious shoulder muscle development. Indeed, as explained in the introduction, the association between bench press and iron cross (correlation coefficient of 0.67) might reflect the muscles contributing to both exercises (pectoralis major, triceps brachii, and latissimus dorsi). As explained in the method, the bench press exercise has been selected due to its good relationship with still rings performance but also because it presents an overall systemic picture of the shoulder's status: “*The strong correlations between the* Bench Press *exercise and all three ring strength elements (though only one was significant) were surprising, since* Bench Press *is rather unspecific at first glance […] the importance of the pectoral muscles for the holding elements on rings could help explain these relationships.*” (Hübner and Schärer 2015). Improving solely the bench press relative strength by exclusively doing this exercise is unlikely to be the best option to perform the iron cross since strength and conditioning need to be harmonious around the shoulder. Similarly, a recent study analyzed the effectiveness of 7 different preconditioning exercises in replicating muscular excitation, coactivation as well as synergies observed during the support scale (Rosaci et al. 2025). Although none of the seven exercises were able to replicate accurately the characteristics of the support scale, the authors recommend performing all of them to specifically target different aims (emphasizing the technic, strengthening the stabilizing muscles of the scapula, strengthening the muscles involved in humeral adduction, strengthening the shoulder flexion action, etc.). Therefore, a high bench press and a low iron cross relative strength could help diagnose weak muscles participating in the iron cross (S. Bernasconi et al. 2004) but not targeted by the bench press (Stastny et al. 2017): biceps brachii and teres major. Additionally, based on the results of Hubner et al., the bench press has shown not only an interesting correlation with the iron cross but also with the support scale (*r* = 0.70) and the swallow (*r* = 0.71). The philosophy behind the present model, namely, the interaction between general and specific strength and conditioning, could be translated to other strength elements.

## 6 Limitations

This study is in the authors' knowledge the first one to investigate the relationship between bench press and iron cross maximal isometric contraction. Nevertheless, some limitations need to be disclosed: Firstly, most gymnasts came to the protocol the day after their competition, which might result in some residual fatigue. Secondly, the handheld dynamometers were 1‐axis sensors, which means that only the orthogonal force to the contact surface was recorded. Additionally, as stated in the method, the granularity of the bench‐press height implies interindividual shoulder and elbow angle variation. These variations could potentially have an implication on the contribution of the different muscles and consequently the force applied to the bar. Despite including solely subjects familiarized with both bench press and iron cross exercise, 95% ICC interval confidence ranged from “Moderate” to “Excellent”. Additional familiarization sessions prior to the protocol might have improved test reliability. Replication studies would also confirm that the present results (i.e., linear threshold between bench press and iron cross relative strength as well as a 170% minimal value in bench press to be able to perform the iron cross) are not due to random sampling. The present protocol took place during a subelite national competition where only one athlete was able to perform the iron cross. Replication with a population composed of a larger proportion of performers would help to unravel the far‐right part of the theoretical model presented in dashed‐line to highlight uncertainty as well as evaluate the generalizability of the current model to elite gymnast. Indeed, the outside envelope presented in Figure 2B suggests that there could be an optimal relative force on bench press to perform the iron cross and that strength in iron cross position could decrease in both directions with either too low or too high force in bench press. Zone number 0 displayed in red on Figure 2C where no gymnasts are able to perform the cross presumably due to a low bench press strength could be symmetrical on the right part. It is possible that a value too high on the bench press (e.g., 300% BW) could be useless, or even detrimental to the performance of the Iron Cross, due to training time requirements as well as gymnast body weight influencing relative strength paramount in gymnastics. Longitudinal follow‐up of gymnasts is currently under investigation to validate the theoretical model provided in the article and include more elite level gymnasts.

## 7 Conclusion

The aim of the study was to analyze the possible interaction between the iron cross and the bench press maximal isometric contraction. The main findings from the present study are twofold: First, the absence of a linear relationship between those two maximal isometric contractions for a large population of subelite gymnasts. Second, the existence of a linear threshold rather than a linear trend between bench press and iron cross (Equation 4). The linear trend evoked in the literature seems to exist solely for the best performers and reflect a homogeneous group of athletes pressing up against their individual theoretical max. Based on the present findings, a theoretical model has been developed to help coaches locate gymnasts and provide individual personalized guidelines to improve the performance of the iron cross depending on the athletes' current general and specific strength status. From the theoretical model, the threshold governing the interaction between those two relative strengths stresses the need to improve the bench press in order to improve the iron cross. Indeed, the absence of gymnasts with a low bench press and a high iron cross relative strength highlights the necessities of the bench press to perform the iron cross. Nevertheless, the important number of gymnasts presenting a high bench press and a low iron cross relative strength emphasize that while being necessary for the iron cross, the bench press is not sufficient.

## Conflicts of Interest

The authors declare no conflicts of interest.
